# Compliance with the “Baby‐friendly Hospital Initiative for Neonatal Wards” in 36 countries

**DOI:** 10.1111/mcn.12690

**Published:** 2018-10-12

**Authors:** Ragnhild Maastrup, Laura N. Haiek, The Neo‐BFHI Survey Group, Welma Lubbe, Deena Yael Meerkin, Leslie Wolff, Kiyoshi Hatasaki, Mona A. Alsumaie, Socorro De Leon‐Mendoza, Yvonne P.M. Ng, Shefaly Shorey, Roxana Conti, Taynara Leme, Edilaine Giovanini Rossetto, Andrea Aldana Acosta, Ana Esther Ortiz Nuñez, Esther Toala, Mirian Elizabeth Ortigoza Gonzalez, Angelika Berger, Yves Hennequin, Anita Pavicic Bosnjak, Hannakaisa Niela‐Vilén, Claire Laurent, Sylvaine Rousseau, Rakel Jonsdottir, Elise M. Chapin, Amanda Smildzere, Rasa Tamelienėd, Raminta Žemaitienė, Maryse Arendt, Mette Ness Hansen, Anette Schaumburg Huitfeldt, Urszula Bernatowicz‐ Łojko, Maria do Céu Barbieri‐ Figueiredo, Ana Paula França, Liubov Abolyan, Irina Pastbina, Carmen Pallás‐Alonso, Maria Teresa Moral‐Pumarega, Mats Eriksson, Renée Flacking, Emily Johnson, Shannon Anderson, Jola Berkman, Diane Boswall, Donna Brown, Julie Emberley, Michelle LeDrew, Maxine Scringer‐Wilkes, Sonia Semenic, Nicole Perriman, Debbie O'Donoghue

**Affiliations:** ^1^ Department of Neonatology Copenhagen University Hospital Rigshospitalet Copenhagen Denmark; ^2^ Ministère de la Santé et des Services sociaux Direction générale de la santé publique Quebec Quebec Canada; ^3^ McGill University, Department of Family Medicine Montreal Quebec Canada; ^4^ St. Mary's Hospital St. Mary's Research Centre Montreal Quebec Canada

**Keywords:** Baby‐friendly Hospital Initiative, breastfeeding, lactation, monitoring, neonatal, preterm

## Abstract

In 2012, the Baby‐friendly Hospital Initiative for Neonatal Wards (Neo‐BFHI) began providing recommendations to improve breastfeeding support for preterm and ill infants. This cross‐sectional survey aimed to measure compliance on a global level with the Neo‐BFHI's expanded Ten Steps to successful breastfeeding and three Guiding Principles in neonatal wards. In 2017, the Neo‐BFHI Self‐Assessment questionnaire was used in 15 languages to collect data from neonatal wards of all levels of care. Answers were summarized into compliance scores ranging from 0 to 100 at the ward, country, and international levels. A total of 917 neonatal wards from 36 low‐, middle‐, and high‐income countries from all continents participated. The median international overall score was 77, and median country overall scores ranged from 52 to 91. Guiding Principle 1 (respect for mothers), Step 5 (breastfeeding initiation and support), and Step 6 (human milk use) had the highest scores, 100, 88, and 88, respectively. Step 3 (antenatal information) and Step 7 (rooming‐in) had the lowest scores, 63 and 67, respectively. High‐income countries had significantly higher scores for Guiding Principles 2 (family‐centered care), Step 4 (skin‐to‐skin contact), and Step 5. Neonatal wards in hospitals ever‐designated Baby‐friendly had significantly higher scores than those never designated. Sixty percent of managers stated they would like to obtain Neo‐BFHI designation. Currently, Neo‐BFHI recommendations are partly implemented in many countries. The high number of participating wards indicates international readiness to expand Baby‐friendly standards to neonatal settings. Hospitals and governments should increase their efforts to better support breastfeeding in neonatal wards.

**Key messages**

- The Neo‐BFHI recommendations were partly implemented in 36 countries with an overall score of 77 out of 100.
- Compliance with the International Code of Marketing of Breast‐milk Substitutes was high in neonatal wards regardless of country's income group.
- Significantly higher compliance was found in high‐income countries for three partial scores: family‐centered care, skin‐to‐skin contact, and breastfeeding/lactation initiation.
- Scores on neonatal wards in hospitals ever‐designated Baby‐friendly were significantly higher than in those never designated.
- The study indicates international readiness for expansion of Baby‐friendly standards to neonatal settings. Hospitals and governments should increase their efforts to protect, promote, and support breastfeeding in preterm and ill infants.

## 1 INTRODUCTION

Globally, an estimated 15 million infants are born prematurely every year (Liu et al., 2016). Breastfeeding is the optimal way of providing infants and young children with the nutrients they need for healthy growth and development, including those who are born preterm or ill (World Health Organization, 2017a). Breastfeeding and breast milk improve short‐ and long‐term outcomes among these vulnerable infants by protecting against serious complications such as sepsis and necrotizing enterocolitis (Henderson, Anthony, & McGuire, 2007). An editorial in a Lancet series on breastfeeding reinforced that no infant or mother should be excluded from breastfeeding promotion activities and called for “a genuine and urgent commitment from governments and health authorities to establish a new normal: where every woman can expect to breastfeed, and to receive every support she needs to do so.” (“Breastfeeding: Achieving the New Normal,” 2016). This commitment should include mothers with infants in the neonatal ward.

Historically, neonatal wards have presented obstacles to successful breastfeeding e.g., mother–infant separation, delayed breastfeeding initiation, and bottle‐feeding (Davis, Mohay, & Edwards, 2003; Maastrup, Bojesen, Kronborg, & Hallstrom, 2012). Preterm and ill infants may not be able to breastfeed right from birth but with a supportive environment can establish exclusive breastfeeding (Maastrup et al., 2014) as recommended for the first 6 months of life (World Health Organization, 2017a). A supportive environment recognizes parents as the most important people in their infants' lives and addresses many of the obstacles present in the neonatal ward.

Since 1991, the WHO/UNICEF's Baby‐friendly Hospital Initiative (BFHI) has provided breastfeeding‐related standards, primarily for maternity wards. Although these include criteria for infants in neonatal care, they are limited in number and scope (World Health Organization/UNICEF, 2009). From 2012 to 2015, a Nordic and Quebec (Canada) working group launched an evidence‐based expansion of the BFHI to neonatal wards (the Neo‐BFHI) of all levels of care. The Neo‐BFHI includes an adaptation of the BFHI's Ten Steps to successful breastfeeding to the special needs of preterm and ill infants, as well as compliance with the International Code of Marketing of Breast‐milk Substitutes and subsequent relevant World Health Assembly resolutions (Code). Three Guiding Principles were added as basic tenets to the Ten Steps (Nyqvist et al., 2012; Nyqvist et al., 2013; Nyqvist et al., 2015; see Table 1). The Neo‐BFHI package can be consulted to obtain detailed information on the background and rationale for the expansion, as well as recommended standards and criteria (Nyqvist et al., 2015). In 2017, six countries reported having a baby‐friendly neonatal certification process separate from the original one (World Health Organization, 2017b). However, there is no information on implementation and certification using the Neo‐BFHI package.

**Table 1:** The Baby‐friendly Hospital Initiative for Neonatal Wards (Neo‐BFHI)

		Indicators (*n*)
Three Guiding Principles		
Guiding principle 1	Staff attitudes towards the mother must focus on the individual mother and her situation.	2
Guiding principle 2	The facility must provide family‐centered care, supported by the environment.	6
Guiding principle 3	The health care system must ensure continuity of care from pregnancy to after the infant's discharge.	3
Expanded Ten Steps to successful breastfeeding		
Step 1	Have a written breastfeeding policy that is routinely communicated to all health care staff.	4
Step 2	Educate and train all staff in the specific knowledge and skills necessary to implement this policy.	5
Step 3	Inform hospitalized pregnant women at risk for preterm delivery or birth of a sick infant about the benefits of breastfeeding and the management of lactation and breastfeeding.	2
Step 4	Encourage early, continuous and prolonged mother‐infant skin‐to‐skin contact/Kangaroo Mother Care.	6
Step 5	Show mothers how to initiate and maintain lactation, and establish early breastfeeding with infant stability as the only criterion.	10
Step 6	Give newborn infants no food or drink other than breast milk, unless medically indicated.	2
Step 7	Enable mothers and infants to remain together 24 hours a day.	3
Step 8	Encourage demand breastfeeding or, when needed, semi‐demand feeding as a transitional strategy for preterm and sick infants.	4
Step 9	Use alternatives to bottle feeding at least until breastfeeding is well established, and use pacifiers and nipple shields only for justifiable reasons.	5
Step 10	Prepare parents for continued breastfeeding and ensure access to support services/groups after hospital discharge.	4
Code	Compliance with the International Code of Marketing of Breast‐milk Substitutes and relevant World Health Assembly resolutions.	7
	Indicators (*N*)	63

Compliance with breastfeeding‐related policies and practices in maternity wards has been measured with varying methods. For example, a Quebec province‐wide measure combined the perspective of staff/managers, mothers, and observers (Haiek, 2012a). However, most surveys have relied on health‐care professional self‐reports, being the most accessible source of information (Centers for Disease Control and Prevention; Crivelli‐Kovach & Chung, 2011; Grizzard, Bartick, Nikolov, Griffin, & Lee, 2006). To date only Denmark and Spain have published countrywide surveys on breastfeeding‐related policies and practices in neonatal wards, both of which are from the manager's perspective (Alonso‐Diaz et al., 2016; Maastrup et al., 2012). Thus, such policies and practices have not been documented in most countries.

The 2018 revision of the BFHI has enlarged its scope to include preterm and ill infants (World Health Organization/UNICEF, 2018). This expanded focus requires the need to examine the current state of breastfeeding support in neonatal wards across different countries in the world. The aim of this study was to assess baseline compliance on a global level with the Neo‐BFHI recommendations. This study was from the perspective of the manager/health care professional in neonatal wards.

## 2 METHODS

### 2.1 Study design

Using a cross‐sectional design, this survey measured neonatal ward compliance with the evidence‐based Neo‐BFHI three Guiding Principles, the expanded Ten Steps, and the Code.

### 2.2 Participants

All neonatal wards, including those providing basic care to the most intensive, were eligible to participate. There were no exclusion criteria, and specifically, the neonatal wards did not need to be aware of the Baby‐friendly programme. Two principal investigators from Denmark and Quebec coordinated the study and invited countries to participate. The first round of invitations included members of a research network; participants from countries in a previous pilot test of the Neo‐BFHI package (Nyqvist et al., 2015); and other individuals who had shown interest in the Neo‐BFHI; 28 countries were invited, and 24 (86%) participated. These included 15 European, 3 Asian, 2 Oceanic, 2 South American, 1 North American, and 1 African country. In order to ensure a more diverse representation, the principal investigators extended the invitation to colleagues during conference presentations and to other breastfeeding‐related professional networks; 39 additional countries were invited of which 12 (31%) participated. The participating 36 countries represent 54% of all those invited. Each participating country/region (with few exceptions) had one or more designated “country survey leaders” who were responsible for recruiting the wards and following up on data collection.

### 2.3 Instrument

Compliance was measured with the Neo‐BFHI's Self‐Assessment questionnaire. The questionnaire was adapted by the principal investigators from the self‐appraisal tool in the Neo‐BFHI package (Nyqvist et al., 2015), which was modelled after the BFHI “Section 4: Hospital Self‐Appraisal and Monitoring” (World Health Organization/UNICEF, 2009). The adaptation consisted of converting existing questions in the self‐appraisal tool into statements. When a question measured two elements (e.g., sound and light in the neonatal environment), it was split in two statements. Most of the yes/no answer choices in the original tool were replaced by a 5‐point Likert scale.

The questionnaire was developed in English and French. It was pilot tested for face and content validity in Quebec, Denmark, the United Kingdom, and France by 11 persons. Thereafter, statements that were hard to measure or repetitive were removed, and those referring to the content of ward protocols were grouped into one statement. With these modifications, the 81 indicators in the self‐appraisal tool were reduced to 63, varying from two to 10 for each of the three Guiding Principles, the Ten Steps, and the Code (see supporting information S1).

Next, the questionnaire was translated into 13 other languages in collaboration with the country survey leaders. The principal investigators were fluent in four languages (English, French, Danish, and Spanish) and had reading skills in four (Italian, Portuguese, Norwegian, and Swedish). These eight languages were the most used. All translations were checked for face validity.

The Neo‐BFHI Self‐Assessment questionnaire was administered via the online software EasyTrial for 11 of the languages. Neonatal wards from nine countries completed the questionnaire on paper (even if some of the languages were available online), and their responses were entered in the online software by their respective country survey leader.

### 2.4 Data collection

The data were collected from February to December 2017. Each participating neonatal ward received one questionnaire. Time needed to complete it was estimated at 1 hr. Participants were instructed to ensure that the questionnaire was answered by the person(s) with the best knowledge of current breastfeeding practices in the ward. The country survey leaders reminded the participants at least three times to complete the questionnaire: 3, 5, and 6 weeks after the initial invitation. All fields in the online questionnaire had to be completed before it could be submitted.

Participating countries were classified as low, middle, and high income using the World Bank Atlas method (The World Bank, 2018).

### 2.5 Statistical analyses

The approach used to assess compliance was based on a methodology used by Haiek (2012a, 2012b) and the Centers for Disease Control and Prevention (2017). Statements measured with 5‐point Likert scales (*None to All* or *Never to Always*) were numerically equivalent to 0–25–50–75–100 points. “Yes” responses were equivalent to 100 points; “No” and “Don't know” to 0 points. In the paper versions, unanswered statements were assigned 0 points. “Don't know” and missing answers did not contribute points because the practice was only considered compliant if the respondent was aware of it.

Most indicators were measured by one statement. Nine were measured by more than one statement, and the points attributed to the indicator were the mean of the points for each statement. Three indicators where graduated into levels; fulfilling the minimum level was regarded as being compliant.

Partial scores refer to each Guiding Principle, Step, and Code. Overall scores refer to a mean or median of the partial scores. The partial scores are used to calculate the overall scores. First, compliance was calculated for each ward as the mean of the points obtained for each indicator measuring the three Guiding Principles, Ten Steps, and the Code, resulting in 14 ward partial scores. The ward overall score was then calculated as the mean of the ward partial scores. An indicator was considered not applicable when the practice could not be measured (e.g., if no breast pumps were available, the indicator for its use was not applicable) and did not contribute to the score.

Second, for each of the 36 countries, the country partial scores were calculated as the median of their ward partial scores, and the country overall score was calculated as the median of their ward overall scores. Finally, the international partial scores were calculated as the median of the country partial scores, and the international overall score as the median of the country overall scores. All scores ranged between 0 and 100. Medians (instead of means) were used for country and international scores, as some countries had very low numbers of participating wards and others had a distribution of scores that violated the assumption of normality.

Descriptive and inferential statistics were used to analyse data. Means are presented with standard deviations and medians with interquartile range. Country and international scores were calculated by level of neonatal care as well as all levels combined; the two‐sample *t* test, one‐way ANOVA, Dunnett's test, and Scheffe test were used to test for differences. A *p* value less than 0.05 was considered statistically significant.

A benchmark report was prepared for each neonatal ward presenting the results for their ward, their country, and international.

### 2.6 Ethics approval and consent to participate

The study was approved by the Research Ethics Committee of St. Mary's Hospital Center, a McGill University teaching hospital in Montreal, Quebec (reference number SMHC # 16‐37). Other countries also sought ethical approval. Given that the survey did not include personally identifiable data and fell into the realm of public health practice, (Hodge Jr, 2005) most countries participated without need of approval from an ethics or data protection committee.

The invitation to participants clarified that answering the questionnaire implied consent to participate. Confidentiality was ensured by allocating to each neonatal ward a unique identification code kept in a separate database and only used to prepare personalized benchmark reports. Results in this paper are reported by country level, to avoid identification of individual wards. Results from countries with two wards are reported without interquartile ranges. Iceland participated with one ward and agreed in reporting their results even though anonymity could not be preserved.

## 3 RESULTS

### 3.1 Participant characteristics

Thirty‐six low‐, middle‐, and high‐income countries from all continents participated in the survey (see Table 2). Twenty‐one countries invited all their neonatal wards, and eight invited all neonatal wards in one or more defined regions in their country, with a mean response rate of 82%. Seven countries invited selected neonatal wards. In total, 917 neonatal wards completed the survey, of which 582 were Level 3 wards. Eighty‐four percent of the wards used either the English questionnaire or a version with the other seven languages understood by the principal investigators. The wards had a mean of 21 neonatal beds. Among participating wards, 35% were in a hospital that was currently or had previously been designated Baby‐friendly. Sixty percent of all respondents stated they would like to obtain or maintain BFHI certification for their neonatal ward by 2019 (see Table 3).

**Table 2:** Characteristics of participating countries

Continent and country	Area covered	Population country/region (millions)	World Bank Country Groups^a^	Eligible wards in country/region (*n*)	Participating wards (n)	Response rate country/region (%)	Language of the questionnaire
Africa (2)							
Gambia^b^	Selected	2	1	Unknown	2	NA	English
South Africa^b^	Regions	56	3	69	33	48	English
Asia (5)							
Israel	Whole country	8.5	4	24	7	29	English
Japan^c^	Selected	127	4	Unknown	23	NA	Japanese
Kuwait^d^	Whole country	4	4	4	4	100	English
Philippines^e^	Regions	103	2	119	67	56	English
Singapore^f^	Whole country	5.6	4	7	7	100	English
Central and South America (6)							
Argentina^g^	Selected	44	3	Unknown	22	NA	Spanish
Brazil^h^	Whole country	208	3	91	51	56	Portuguese
Colombia^i^	Region	8	3	35	22	63	Spanish
Ecuador^j^	Selected	16	3	Unknown	11	NA	Spanish
Panama	Whole country	4	3	5	5	100	Spanish
Paraguay	Whole country	6.7	3	41	41	100	Spanish
Europe (20)							
Austria	Whole country	8.7	4	21	15	71	English
Belgium	Whole country	11.4	4	19	19	100	English/French
Croatia	Whole country	4.2	3	13	13	100	Croatian
Denmark	Whole country	5.7	4	19	19	100	Danish
Estonia	Whole country	1.3	4	6	5	83	Estonian
Finland	Whole country	5.5	4	23	18	78	Finnish
France^d^^,^ k	Regions	7	4	34	27	79	French
Iceland	Whole country	0.3	4	1	1	100	English
Italy^l^	Selected	61	4	Unknown	47	NA	Italian
Latvia	Whole country	2	4	6	6	100	English
Lithuania	Whole country	2.8	4	7	7	100	Lithuanian
Luxembourg	Whole country	0.6	4	2	2	100	French
Norway^d^	Whole country	5.2	4	20	20	100	Norwegian
Poland^m^	Region	2	4	19	19	100	Polish
Portugal^n^	Regions	7.5	4	30	19	63	Portuguese
Russia^o^	Selected	144	3	Unknown	60	NA	Russian
Slovenia^p^	Selected	2.1	4	Unknown	2	NA	English
Spain	Whole country	47	4	153	137	90	Spanish
Sweden	Whole country	9.9	4	39	34	87	Swedish
UK (6 regions)^d^^,^ q	Regions	15	4	56	34	61	English
North America (1)							
Canada^r^	Regions	19	4	92	89	97	French/English
Oceania (2)							
Australia	Whole country	24	4	34	14	41	English
New Zealand	Whole country	4.7	4	23	15	65	English
Total					917		

*Note*. NA: nonapplicable. Selected: The invited wards were selected among neonatal wards in the country and the population size refers to the whole country.

a World Bank Country Groups 1 = low‐income, 2 = lower middle‐income, 3 = upper middle‐income, 4 = high‐income.

Invited two wards.

b Invited after group or individual ethical approval.

c Invited 23 wards, every BFHI hospitals with a neonatal ward.

d Country has a Baby‐friendly neonatal certification process separate from the original one.

e One region: Davao, and Philippine Society of Newborn Medicine.

f All public hospitals invited.

g Invited 32 wards from network, mainly level 3, many provinces represented.

h Invited after individual ethical approval.

i One region: Bogota city.

j Invited 12 wards.

k Hauts de France and Marseille city, one out of 13 regions and one city.

l Invited 47 wards. All wards invited in two regions and one city (Emilia Romania, Toscany and Milan). Rest of the country invited by network. Hospitals from 15 of 20 Regions participated, mainly level 3.

m One region: Kuyavian‐Pomeranian Voivodeship.

n Six of seven regions: Madeira, Azores, North, Centre, Alentejo and Algarve.

o Invited 63 wards, One region: Archangelsk Region (1.2 mill) and BFHI network (including 18 of 85 regions).

p Invited two wards, Neo‐BFHI interested.

q Five regions: East of England, South West, NW London, NC London, Northern.

r Seven regions: Alberta, British Columbia, New Brunswick, Newfoundland, Nova Scotia, Prince Edward Island, and Quebec.

**Table 3:** Characteristics of participating neonatal wards

	Mean (SD) or median (IQR)	Number of wards (%)
Number of beds, mean (SD)	21 (19)	
Number of infants in the ward^a^, Mean (SD)	16 (17)	
Ward has an early discharge programme for preterm infants with nasogastric tube in order to establish breastfeeding at home		168 (19)
Ward has Kangaroo Mother Care programme for preterm infants with early discharge and follow‐up		239 (26)
Ward has access to banked or donor human milk		408 (45)
Hospital has breastfeeding related committees		493 (54)
Neonatal ward in hospital ever designated “Baby‐friendly”		317 (35)
Respondent's intention to obtain/maintain BFHI for neonatal ward		553 (60)
Level of neonatal care (definitions in foot notes)^b^		
Level of care 1^c^		151 (16)
Level of care 2^d^		184 (20)
Level of care 3A^e^		185 (20)
Level of care 3B^f^		344 (38)
Level of care 3C^g^		53 (6)
Type of ward^b^		
Exclusive neonatal		534 (59)
Mixed neonatal‐maternity		244 (27)
Mixed neonatal‐paediatric		90 (10)
Other		40 (4)
Type and number of staff that have direct responsibility for assisting mothers in the neonatal ward with lactation, breastfeeding and infant feeding^h^		
Nurses/midwives working primarily in neonatal ward, Median (IQR)	25 (13–45)	877 (97)
Lactation consultants, Median (IQR)	2 (1–3)	392 (43)
Physicians, Median (IQR)	7 (4–13)	732 (81)
Dieticians/Nutritionists		219 (24)
Occupational therapists/Speech therapists		231 (25)
Lay support persons/peer counsellors		68 (7)
Other		60 (7)
No staff responsible		10 (1)
Questionnaire answered by^h^		
Head Nurse		319 (35)
Breastfeeding Staff		256 (28)
Physicians		242 (27)
Other		304 (33)
Questionnaire answered by more than one person		175 (22)

*Note*. IQR = interquartile range, SD = standdard deviation

a Calculated for the day before answering the questionnaire.

b Because the responses to the statement are mutually exclusive, the sum of results is equal to 100%.

c Level 1 = Basic care of stable infants born at 35 to less than 37 weeks gestation.

d Level 2 = Specialty care of infants born at least 32 weeks gestation or 1,500 grams, with possibility of brief mechanical ventilation or CPAP.

e Level 3A = Subspecialty intensive care of infants born at least 28 weeks gestation or 1,000 grams with possibility of mechanical ventilation.

f Level 3B = Subspecialty intensive care of infants born at less than 28 weeks gestation or 1,000 grams, with possibility of advanced respiratory support, and access to paediatric surgical specialist.

g Level 3C = As level 3B but including extracorporeal membrane oxygenation and surgical repair of complex congenital cardiac malformations.

h Because the statement allowed more than one answer, the sum of results is equal or greater to 100%.

### 3.2 Compliance scores

**Figure 1:**
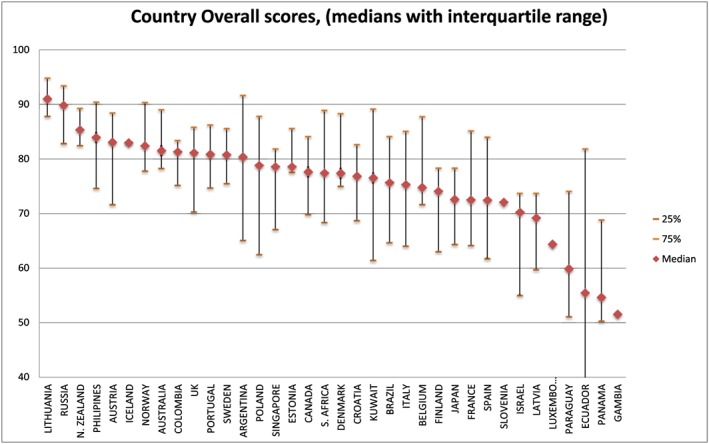
Country overall scores. Medians with interquartile range

The international overall score was 77 with country overall scores ranging from 52 in Gambia to 91 in Lithuania (see Table 4 and Figure 1). Even though there were no significant differences in country overall scores between high‐income and low, middle‐income countries, we found significantly higher country partial scores in high‐income countries for Guiding Principle 2 (median 83, 95% CI [79.1, 87.7] vs. 71, 95% CI [63.8, 78.3], *p* = 0.0023); Step 4 (median 79, 95% CI [74.0, 83.8] vs. 63, 95% CI [48.3, 77.7], *p* = 0.0091); and Step 5 (median 87, 95% CI [84.5, 89.7] vs. 81, 95% CI [75.0, 87.3], *p* = 0.0316). Neonatal wards situated in a hospital ever‐designated Baby‐friendly had significantly higher ward overall scores (mean 83.2, 95% CI [82.0, 84.3]) than wards in non‐BFHI hospitals (mean 72.3, 95% CI [71.1, 73.4], *p* < 0.0001). This was also true for all partial scores (Table 5).

**Table 4:** Summary of partial and overall scores for country and international level

		Country Partial Scores														Country Overall Scores		
		GP 1	GP 2	GP 3	Step 1	Step 2	Step 3	Step 4	Step 5	Step 6	Step 7	Step 8	Step 9	Step 10	Code	Median	25% quartile	75% quartile
Continent and country	Number of indicators (*N* = 63) / Number of wards (*N* = 917)	2	6	3	4	5	2	6	10	2	3	4	5	4	7			
Africa																		
Gambia	2	75	51	50	0	31	0	56	65	100	50	91	98	13	43	**52**		
South Africa	33	100	82	92	75	84	81	71	90	88	58	81	85	75	82	**77**	68	89
Asia																		
Israel	7	100	72	75	0	47	50	68	80	100	50	69	70	75	68	**70**	55	74
Japan	23	100	69	92	92	63	38	62	83	75	42	75	50	72	96	**73**	64	78
Kuwait	4	94	52	79	96	88	88	49	90	88	54	81	83	78	98	**77**	61	89
Singapore	7	100	76	75	100	95	75	61	94	75	33	50	75	81	100	**79**	67	82
Philipines	67	100	78	92	100	93	81	80	80	100	67	88	100	81	96	**84**	75	90
Central & South America																		
Argentina	22	81	77	92	92	83	38	79	88	94	100	84	75	75	96	**80**	65	92
Brazil	51	88	75	83	75	80	63	71	80	88	67	88	95	69	82	**76**	65	84
Colombia	22	100	81	75	100	80	88	88	76	75	67	69	55	81	96	**81**	75	83
Ecuador	11	88	64	67	0	38	25	54	72	100	58	69	70	59	68	**55**	35	82
Panama	5	75	54	100	25	63	50	0	70	75	17	50	50	50	96	**55**	50	69
Paraguay	41	75	61	83	0	48	38	62	75	75	67	75	75	56	64	**60**	51	74
Europe																		
Austria	15	100	93	100	58	78	88	88	90	88	67	94	80	88	96	**83**	72	88
Belgium	19	100	86	75	71	75	88	86	90	88	92	75	80	75	82	**75**	72	88
Croatia	13	100	67	83	100	90	63	50	93	88	33	81	75	88	82	**77**	69	83
Denmark	19	100	92	92	58	75	63	93	88	88	100	81	85	69	86	**77**	75	88
Estonia	5	100	96	100	83	70	69	87	95	75	67	88	90	56	68	**79**	78	86
Finland	18	88	73	83	75	78	13	84	80	88	92	78	63	72	75	**74**	63	78
France	27	100	86	83	0	59	50	82	88	63	92	81	80	94	82	**72**	64	85
Iceland	1	100	89	83	83	75	63	96	78	100	92	63	75	69	96	**83**	83	83
Italy	47	88	83	92	75	84	63	73	88	88	67	75	65	75	86	**75**	64	85
Latvia	6	100	91	92	17	68	13	73	78	75	63	88	69	38	84	**69**	60	74
Lithuania	7	100	93	100	100	98	100	85	100	100	100	100	81	84	100	**91**	88	95
Luxembourg	2	88	80	79	33	56	19	84	83	50	83	56	58	69	64	**64**		
Norway	20	94	88	92	75	84	81	79	91	88	100	84	83	75	98	**82**	78	90
Poland	19	100	85	75	67	88	50	65	93	75	100	88	75	56	82	**79**	62	88
Portugal	19	100	85	92	75	73	50	80	90	100	75	94	80	75	71	**81**	75	86
Russia	60	100	79	100	100	95	88	81	93	88	100	94	94	94	96	**90**	83	93
Slovenia	2	88	71	96	50	67	88	60	79	81	46	69	70	78	84	**72**		
Spain	137	88	82	83	75	60	50	82	80	75	67	81	75	75	68	**72**	62	84
Sweden	34	88	93	88	71	63	63	90	90	75	100	94	88	75	82	**81**	75	86
UK	34	88	85	75	75	76	63	83	90	81	83	88	85	69	96	**81**	70	86
North America																		
Canada	89	88	88	83	75	70	50	85	85	75	100	75	75	81	82	**78**	70	84
Oceania																		
Australia	14	100	88	96	71	76	75	83	88	88	100	81	80	88	95	**82**	78	89
New Zealand	15	100	92	92	83	93	25	83	95	88	100	88	90	94	100	**85**	82	89

		International Partial Scores														International Overall Scores		
		GP 1	GP 2	GP 3	Step 1	Step 2	Step 3	Step 4	Step 5	Step 6	Step 7	Step 8	Step 9	Step 10	Code	Median	25% quartile	75% quartile
International score, all levels of care		**100**	**82**	**85**	**75**	**76**	**63**	**80**	**88**	**88**	**67**	**81**	**78**	**75**	**84**	**77**	**72**	**81**
25% quartile		88	73	79	54	63	44	64	80	75	58	75	70	69	79	72		
75% quartile		100	88	92	88	84	81	84	90	88	100	88	85	81	96	81		

*Note*. The colour code in the countries' names indicates the following: green, all wards of the country/region were invited, response rate above or equal to 85%; yellow, all wards in the country/region were invited, response rate below 85%; and red, selected wards of a country/region were invited, irrespective of response rate. The numbers in bold indicate the main results for the median country overall scores and the International partial scores “International score, All levels of care.”

**Table 5:** Comparison of ward partial scores in ever versus never BFHI‐designated hospitals

	Ward in ever BFHI designated hospital (*N* = 317)	Ward in never BFHI designated hospital (*N* = 535)	
GP/Step/Code	Mean (SD)	Mean (SD)	*p* value
GP1	92 (12)	88 (13)	0.0001
GP2	81 (14)	78 (16)	0.0038
GP3	88 (14)	82 (18)	<0.0001
Step 1	87 (22)	54 (41)	<0.0001
Step 2	83 (19)	65 (24)	<0.0001
Step 3	67 (33)	52 (35)	<0.0001
Step 4	77 (19)	72 (23)	0.0004
Step 5	88 (12)	81 (15)	<0.0001
Step 6	84 (18)	77 (20)	<0.0001
Step 7	78 (26)	72 (27)	0.0032
Step 8	85 (16)	75 (20)	<0.0001
Step 9	82 (19)	71 (21)	<0.0001
Step 10	80 (18)	71 (23)	<0.0001
The Code	90 (14)	72 (23)	<0.0001
Overall mean	83 (10)	72 (13)	<0.0001

*Note*. BFHI: Baby‐friendly Hospital Initiative; GP: guiding principle; SD: standard deviation.

There were no significant differences in the country overall scores between the countries with response rates of at least 85% versus those with less, or between countries who invited all wards versus those who selected their wards. There were no significant differences in overall international scores between Levels 1, 2, and 3 of neonatal care.

The three Guiding Principles had generally high international partial scores (100, 82, and 85; Table 4 and supporting information S2). Twenty countries had a partial score of 100 for Guiding Principle 1 based on indicators about treating mothers with sensitivity, empathy, and respect for their maternal role and supporting them in making informed decisions about milk production, breastfeeding, and infant feeding. The steps with the highest international partial scores were Steps 5 and 6 (both 88; Table 4). Among 10 indicators measuring initiation of breastfeeding and breastmilk expression (Step 5), the one best implemented stated “Infant stability is the only criterion for early initiation of breastfeeding.” This indicator was answered “Yes” by 80% of the wards. In Step 6, the indicator “Infants in your ward are fed only breast milk, unless there are acceptable medical reasons to use breastmilk substitutes” was answered “Many” or “All” (infants) by 80% of the wards.

Step 3 about antenatal information had the lowest international partial score (63), followed by Step 7 about rooming‐in (67). Both steps had very large variations in country partial scores, ranging from 0 to 100 for Step 3 and from 17 to 100 for Step 7. For Step 3, only 24% of wards in hospitals that had hospitalized pregnant women reported always visiting the mother antenatally to offer her information about breastfeeding and lactation. Although 10 countries had a partial score of 100 for Step 7, many countries had restrictions on mothers' presence beside their infant's bed and did not provide mothers the possibility of rooming‐in on the ward or elsewhere in the hospital. In fact, mothers were able to sleep in the same room as their infants for the whole hospital stay in only 18% of the neonatal wards. Step 7 had the lowest scores in Africa, Central & South America, and Asia, as well as lower scores in Southern European compared with Northern European countries.

Step 4 (skin‐to‐skin contact) had an international partial score of 80 with large variations between countries. Nevertheless, 96% of the wards reported that infants were placed in skin‐to‐skin contact with mothers/fathers. Stable infants were allowed to remain skin‐to‐skin for as long and as often as the parents were able and willing in 84% of the wards, but in very few wards (2%), the daily length of skin‐to‐skin contact for stable preterm infants was in general more than 20 hr/day. In 55% of the wards, the estimated daily duration was more than 4 hr. Wards with a Kangaroo Mother Care programme (239) had significantly higher Step 4 partial ward scores than wards with no programme (median 79, 95% CI [77.2, 81.7] vs. 72, 95% CI [70.2, 73.6], *p* < 0.0001).

The Code had an international partial score of 84. Twenty‐three percent of the wards had 100% compliance with the Code. In fact, when considering all 63 indicators, two Code indicators were among the four most highly implemented: 90% of the wards kept infant formula cans and prepared bottles out of view unless in use, and 85% of the wards refrained from promoting breastmilk substitutes, bottles, teats, or pacifiers.

## 4 DISCUSSION

This is the first survey measuring compliance with Neo‐BFHI policies and practices in neonatal wards in countries from all continents. Reported overall compliance with the Neo‐BFHI standards was generally high. All 36 participating countries obtained an overall score higher than 50, demonstrating that all countries implemented the Neo‐BFHI recommendations to some extent. It has previously been found that the original BFHI programme is doable and adaptable in a wide variety of cultural and socio‐economic settings (Saadeh & Casanovas, 2009), but global implementation at the health care facility level is presently unknown (World Health Organization, 2017b; World Health Organization/UNICEF, 2018).

Higher scores obtained by neonatal wards in hospitals ever‐designated Baby‐friendly may be related to the fact that BFHI certification includes some practices related to neonatal care, but it may also demonstrate that maternity and neonatal wards do not operate in isolation (Taylor, Gribble, Sheehan, Schmied, & Dykes, 2011) and the certification process in one ward may have a beneficial effect on the revision of policies and practices of the other one (Alonso‐Diaz et al., 2016). Even though only four countries in the present survey had a Baby‐friendly neonatal certification process separate from the original one, 60% of the respondents expressed intentions to obtain or maintain BFHI certification for their neonatal ward.

It is heartening that high compliance was reported by health care professionals for the three Guiding Principles. Still, we recognize that given the subjective nature of their responses, obtaining the additional perspective of the mother may provide a more holistic picture. It is also noteworthy that the concept of “Infant stability as the only criterion for early initiation of breastfeeding” (at the breast) is globally implemented. This marks an important shift in practice, as preterm infants were traditionally prevented from feeding at the breast until they reached a certain postmenstrual age or weight (Wamback & Riordan, 2016).

We found overall low implementation for Step 7 about enabling mother's presence including the possibility of sleeping by or close to their infants, but with large variations between countries. Restrictions in parents' presence beside their infant in neonatal wards have decreased in the last decades (Davis et al., 2003). However, studies continue to document differences between countries, for example, with more restrictions noted in Southern than Northern Europe (Alonso‐Diaz et al., 2016; Greisen et al., 2009; Maastrup et al., 2012; Pallas‐Alonso et al., 2012), indicating more efforts are required to protect the rights of infants to be cared for by their parents (Office of the United Nations High Commissioner for Human Rights, 1989). Challenges involved in avoiding mother–infant separations are well recognized (Flacking et al., 2012); it has previously been found that Step 7 was one of the least implemented steps in maternity wards (Haiek, 2012a).

Almost every ward in the survey had implemented skin‐to‐skin contact to some extent. This seems to indicate that this life‐saving and breastfeeding‐promoting practice is slowly changing from “nice to do” to “need to do” but there is still room for improvement in implementing early, prolonged, and continuous skin‐to‐skin care (World Health Organization, 2015; World Health Organization/UNICEF, 2018). Despite being an effective low‐cost intervention (Conde‐Agudelo & Diaz‐Rossello, 2016), Step 4 had significantly higher scores in high‐income countries. Noteworthy, Colombia, where skin‐to‐skin care originated, had among the best scores for this step. Although mothers and staff both value skin‐to‐skin contact, staff capacity, staff breastfeeding knowledge, their concerns about time and safety, especially in the neonatal ward, may hinder its implementation (Olsson et al., 2012; World Health Organization, 2017a) as could organizational culture and space‐architectural constraints. The fact that Guiding Principle 2 had higher scores in high‐income countries could be due to similar issues.

As in the original BFHI, we restricted the Code‐related indicators to those involving health facilities. It is encouraging that compliance with the Code was high in the present survey and several of the indicators used to measure it were among the best implemented. This finding underscores the concept that the introduction of the BFHI has led to positive changes in health professionals' attitudes towards breastfeeding protection. Yet a recent report documented that other aspects related to the adoption of legal measures to implement the Code—and the mechanisms to monitor and enforce them—are lacking (World Health Organization/UNICEF/IBFAN, 2018). These may negatively influence health professionals in neonatal wards and the families they care for.

### 4.1 Strengths and limitations

The strengths of this study are the global representation of countries as well as the high number of participating wards. Also, 13 participating countries had 100% response rates. This demonstrates the feasibility of integrating neonatal ward self‐assessments into monitoring systems for Baby‐friendly care, one of the management procedures reaffirmed in the 2018 BFHI revision for both the country and health care facility level (World Health Organization/UNICEF, 2018). From a global health perspective, providing wards with individualized benchmark reports may stimulate quality improvement efforts and facilitate translation of the evidence‐based Neo‐BFHI guidelines into practice.

A limitation of the study is the selection of countries via convenience sampling. Although no country was excluded, the networks used to recruit them had an overrepresentation of high‐income countries, and many of the low‐income countries contacted did not participate, which may hinder the generalization of the results. Also, seven countries did not invite all their wards. The questionnaires in Finnish, Estonian, Lithuanian, Polish, Croatian, Russian, and Japanese, used by 14% of the responders, were not back‐translated. All the country survey leaders who did the translations were familiar with the BFHI terminology.

The study is also limited by the use of health care professional self‐reports. It has been shown that compliance with BFHI standards was significantly higher when reported by staff/managers than parents themselves (Haiek, 2012a). Still, health care professional self‐reports remain one of the most accessible sources of information to measure compliance, and comparison of the present results was done with such studies (Alonso‐Diaz et al., 2016; Grizzard et al., 2006; Maastrup et al., 2012).

## 5 CONCLUSION

An international Neo‐BFHI compliance score of 77 out of 100 and country scores higher than 50 for all 36 participating countries demonstrate that neonatal wards around the world are working to support breastfeeding.

Widespread interest from respondents in obtaining BFHI certification for their neonatal ward calls for key players in hospitals and governments to fully integrate the BFHI into neonatal wards. We welcome that the completion of this large international survey comes at the same time as the publication of the 2018 WHO/UNICEF revision, which enlarges the scope of the BFHI to include preterm and ill infants and achieve a new normal where mothers in the neonatal ward can expect to breastfeed and receive the support they need to do so.

Further research should include parents' perspective, ensure participation of more low‐income countries, and explore the effect of implementing the Neo‐BFHI on breastfeeding outcomes.

### 5.1 Availability of data

The datasets used and analysed during the current study are available from the corresponding author on reasonable request for scientific use.

## CONFLICTS OF INTEREST

The authors declare that they have no conflicts of interest.

## CONTRIBUTIONS

RM and LNH (principal investigators) developed the concept and design of the study, enrolled the countries, analysed and interpreted data, supported country survey leaders, and drafted the article. RM, LNH, CL, and SSe developed the questionnaire. RM, LNH, CL, EJ, and SSe pilot tested the questionnaire. RM, LNH, APF, APB, ASH, CL, CPA, EC, EGR, HNV, KH, LA, MCBF, MNH, RF, RT, RŽ, SSe, and UBŁ translated the questionnaire. All authors contributed to the recruitment of participating wards, collected data, revised the manuscript critically, and approved the final version.

## Supporting information

Data S1 Supporting information

Data S2 International Partial Scores. Medians with interquartile range

## Africa

Welma Lubbe, PhD, (North‐West University, School of Nursing Science, Potchefstroom, South Africa).

## Asia

Deena Yael Meerkin, RN, (Shaarei Zedek Medical Center, Dept. of Neonatology, Jerusalem, Israel).

Leslie Wolff, CNM, (Ichilov Medical Center, Dept. of Neonatology, Tel Aviv‐Yafo. Israel).

Kiyoshi Hatasaki, PhD, (Toyama Prefectural Central Hospital, Dept. of Neonatology, Toyama‐ken, Japan).

Mona A. Alsumaie, MB BCh, (Public Authority of Food and Nutrition, MOH, Community Nutrition Education and Promotion Dept, Sabah Alsalem, Kuwait).

Socorro De Leon‐Mendoza, MD, (Kangaroo Mother Care Foundation Phil., Inc. Parañaque City, Metro Manila, The Philippines).

Yvonne P.M. Ng, MBBS, (Khoo Teck Puat‐ National University Children's Medical Institute, National University Hospital, Dept. of Neonatology, Singapore).

Shefaly Shorey, PhD, (National University of Singapore, Alice Lee Centre for Nursing Studies, Yong Loo Lin School of Medicine, Level 2, Clinical Research Centre, Singapore).

## Central & South America

Roxana Conti, MD, (Promotion and Protection of Health Unit, Hospital Materno Infantil Ramón Sardá, Ciudad Autónoma de Buenos Aires, Argentina).

Taynara Leme, RN, (State University of Londrina, Health Science Center, Vila Operária, Londrina, Brazil).

Edilaine Giovanini Rossetto, PhD, (State University of Londrina, Health Science Center, Vila Operária, Londrina, Brazil).

Andrea Aldana Acosta, PhD, (Fundación Canguro/Universidad Piloto de Colombia, Bogota, Colombia).

Ana Esther Ortiz Nuñez, MD, (Hospital Los Ceibos del IESS, Dept. of Neonatology, Guayaquil, Ecuador).

Esther Toala, MD, (Hospital General, Dept. of Neonatology, Panama, Panama).

Mirian Elizabeth Ortigoza Gonzalez, MD, (Ministry of Public Health and Social Welfare, Dept. of Childhood and Adolescence. Dept. of Breastfeeding, Asunción, Paraguay).

## Europe

Angelika Berger, MD, (Medical University Vienna, Dept. of Pediatrics and Adolescent Medicine, Vienna, Austria).

Yves Hennequin, MD, (Hopital Universitaire des Enfants Reine Fabiola, Dept. of Neonatology, Brussels, Belgium).

Anita Pavicic Bosnjak, PhD, (University Hospital Sv. Duh Croatia, Dept. of Neonatology, Department of Obstetrics and Gynecology Medical School University of Zagreb, Zagreb, Croatia).

Hannakaisa Niela‐Vilén, PhD, (University of Turku, Dept. of Nursing Science, Turku, Finland):

Claire Laurent, MD, (Baby‐friendly Hospital Initiative (IHAB) France), Rives en Seine, France).

Sylvaine Rousseau, MD, (Hospital Roubaix, Dept. of Neonatology, Roubaix, France).

Rakel Jonsdottir, RN, (University Hospital Landspitali, Dept. of Neonatology, Reykjavik, Iceland).

Elise M. Chapin, M.Ed., (Italian National Committee for UNICEF (Insieme per l'Allattamento), Rome, Italy).

Amanda Smildzere, MD, (Riga Children University Hospital, Dept. of Neonatology, Rīga, Latvia).

Rasa Tamelienė, PhD, (Lithuanian University of Health Sciences, Dept. of Neonatology, Kaunas, Lithuania).

Raminta Žemaitienė, MD, (Lithuanian University of Health Sciences, Dept. of Neonatology, Kaunas, Lithuania).

Maryse Arendt, IBCLC, (Luxemburg UNICEF, Baby‐friendly Hospital Initiative, Luxemburg, Luxemburg).

Mette Ness Hansen, MPH, (Oslo University Hospital, Rikshospitalet, Division of Gynaecology and Obstetrics, Norwegian National Advisory Unit on Breastfeeding, Oslo, Norway).

Anette Schaumburg Huitfeldt, RM, (Oslo University Hospital, Rikshospitalet, Division of Gynaecology and Obstetrics, Norwegian National Advisory Unit on Breastfeeding, Oslo, Norway).

Urszula Bernatowicz‐ Łojko, MD, (Provincial Polyclinical Hospital in Toruń, Dept. of Neonatology, Regional Human Milk Bank, Toruń, Poland).

Maria do Céu Barbieri‐ Figueiredo, PhD, (Nursing School of Porto, CINTESIS‐ESEP, Porto, Portugal).

Ana Paula França, PhD, (Nursing School of Porto, Porto, Portugal).

Liubov Abolyan, PhD, (I.M. Sechenov First Moscow State Medical University of the Ministry of Health of the Russian Federation, Dept of Public Health, Moscow, Russia).

Irina Pastbina, MD, (Ministry of health service of the Arkhangelsk region, Dept. of Children's medical care and obstetrics service, Arkhangelsk, Russia).

Carmen Pallás‐Alonso, PhD, (Baby‐friendly Hospital Initiative, Spain and Hospital 12 de Octubre, Dept. of Neonatology, Madrid, Spain).

Maria Teresa Moral‐Pumarega, PhD, (Hospital 12 de Octubre, Dept. of Neonatology, Madrid, Spain).

Mats Eriksson, PhD, (Örebro University, School of Health Sciences, Örebro, Sweden).

Renée Flacking, PhD, (Dalarna University, School of Education, Health and Social Studies, Falun, Sweden).

Emily Johnson, BSc Joint Hons Speech and psychology, (Great Ormond Street Hospital NHS Foundation Trust, Speech and Language Therapy Dept., London, United Kingdom).

## North America

Shannon Anderson, MN, (Covenant Health, Edmonton, Alberta, Canada).

Jola Berkman, RN, (Perinatal Services of British Columbia, West Vancouver, British Columbia, Canada):

Diane Boswall, RN, (Public Health and Children's Developmental Services, Health PEI, Charlottetown, Prince Edward Island, Canada).

Donna Brown, RN, (HHN Baby‐Friendly Initiative, Fredericton, New Brunswick, Canada).

Julie Emberley, MD, (Memorial University, Dept of Pediatrics, St. John's, Newfoundland, Canada).

Michelle LeDrew, Master of Nursing, (IWK Health Centre, Nova Scotia, Canada).

Maxine Scringer‐Wilkes, RN, (Alberta Health Services, Alberta Children's Hospital, Calgary, Alberta, Canada).

Sonia Semenic, PhD, (Ingram School of Nursing, McGill University, Montreal, Quebec, Canada).

## Oceania

Nicole Perriman, RM, (Baby‐friendly Hospital Initiative, Australia and Australian College of Midwives, ACT, Australia).

Debbie O'Donoghue, RN, (Christchurch Women's Hospital, Dept. of Neonatology, Canterbury District Health Board, Christchurch, New Zealand).
