# Supplementary material for: Compliance with the “Baby‐friendly Hospital Initiative for Neonatal Wards” in 36 countries
Source: Matern Child Nutr. 2018 Oct 12;15(2):e12690. doi: 10.1111/mcn.12690 (PMC6586157; doi:10.1111/mcn.12690)
Supplement: Supplementary file 1 — Data S1 Supporting information [file MCN-15-e12690-s001.pdf]

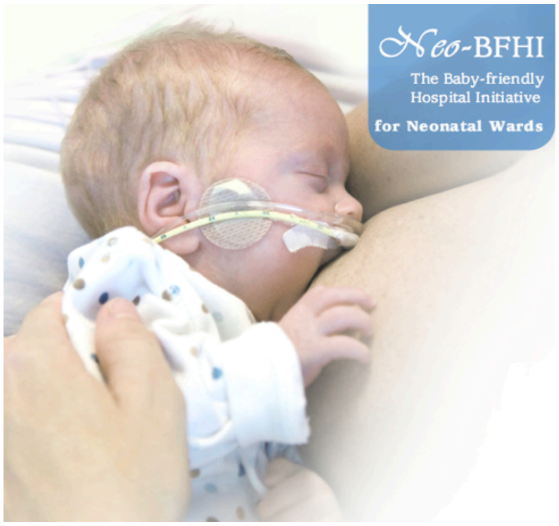

Neo-BFHI Self-Assessment questionnaire

International survey of breastfeeding-related practices and policies in neonatal wards

Questionnaire

(from on-line software)

2017

Guiding principle 1

Staff attitudes toward the mother must focus on the individual mother and her situation.

	Indicators	Options				
GP1_1	**GP1_1** The clinical staff treat mothers with sensitivity, empathy and respect for their maternal roles.	Never	Rarely	Half the time	Frequently	Always
GP1_2	**GP1_2** The clinical staff support mothers in making informed decisions about milk production, breastfeeding and infant feeding.	Never	Rarely	Half the time	Frequently	Always

Note: These indicators apply to all infants admitted to your neonatal ward, whether they are breastfed or not.

Guiding principle 2

The facility must provide family-centered care, supported by the environment.

	Indicators	Options				
GP2_1	**GP2_1** The clinical staff know how family-centered care is integrated in their neonatal ward. Central aspects of family-centered care are respect and dignity, information sharing, participation, and collaboration.	None	Not many	About half	Many	All
GP2_2 (Mean of 2 responses)	**GP2_2** Your neonatal ward is open 24 hours a day, 7 days a week, without restrictions:					
	**GP2_2a** To mothers.	No	Yes			
	**GP2_2b** To fathers/partners.					
GP2_3	**GP2_3** Parents begin to participate in their infants' care within the first 24 hours after the birth.	None	Not many	About half	Many	All
GP2_4 (Best of 3 responses)	**GP2_4** Mothers are able to rest by their infants' bedside in the neonatal ward:					
	**GP2_4a** On a bed/mattress.	Never	Rarely	Half the time	Frequently	Always
	**GP2_4b** On a chair with armrests or on a reclining chair.					
	**GP2_4c** On a chair without armrest.					
GP2_5 (Best of 3 responses)	**GP2_5** Mothers are able to eat close to your neonatal ward (no matter who provides the food).					
	**GP2_5a** Able to eat in the ward.	Never	Rarely	Half the time	Frequently	Always
	**GP2_5b** Able to eat very close to the ward (5 minutes walking distance or less).					
	**GP2_5c** Able to eat close to the ward (6 to 10 minutes walking distance).					
GP2_6 GP2_7 GP2_8 (Mean of 3 responses)	The next statements refer to the environment in the neonatal ward.					
	**GP2_6** The lighting in your neonatal ward is individualized for each infant.	No	Yes			
	**GP2_7** The sound level in your neonatal ward is low.	Never	Rarely	Half the time	Frequentl y	Always
	**GP2_8** The environment in your neonatal ward allows mothers to have privacy.					

Note 1: These indicators apply to all infants admitted to your neonatal ward, whether they are breastfed or not.

Note 2: For indicators GP2_4 and GP2_5, if the response to "a" is Always, "b" and "c" are not asked, and if the answer to "b" is Always, "c" is not asked.

Guiding principle 3

The health care system must ensure continuity of care from pregnancy to after the infant's discharge.

	Indicators	Options				
GP3_1	**GP3_1** Your neonatal ward collaborates with other wards that care for mothers and infants to ensure that the support for lactation, breastfeeding and infant feeding is consistent from one ward to the other.	Never	Rarely	Half the time	Frequently	Always
GP3_2	**GP3_2** The clinical staff know about the infants' medical condition and current care plan (including strategies to support lactation, breastfeeding and infant feeding).	Never	Rarely	Half the time	Frequently	Always
GP3_3	**GP3_3** Information regarding the current situation and plan for lactation, breastfeeding and feeding support is included in reports from your neonatal ward when infants are transferred to the next phase of care.	Never	Rarely	Half the time	Frequently	Always

Note: These indicators apply to all infants admitted to your neonatal ward, whether they are breastfed or not.

Step 1 (Neonatal expansion)

Have a written breastfeeding policy that is routinely communicated to all health care staff.

Original BFHI Step 1: Same wording for the original BFHI and the Neo-BFHI.

	Indicators	Options
S1_1	**S1_1** Your hospital has a written breastfeeding/infant feeding policy.	No Yes Don't know
S1_2 (Mean of 3 responses)	**S1_2** The policy includes:	
	**S1_2a** The Three Guiding Principles as stated previously.	No Yes Don't know
	**S1_2b** The Ten Steps as they apply to neonatal care.	
	**S1_2c** The International Code of Marketing of Breast milk Substitutes as it applies to neonatal wards.	
S1_3	**S1_3** The breastfeeding/infant feeding policy is available so all staff who take care of mothers and babies can refer to it.	No Yes
S1_4<br>S1_5<br>(Mean of 2 responses)	**S1_4** A written summary or visual image of the policy is posted or displayed in your neonatal ward.	No Yes
	**S1_5** The summary of the policy is posted in the language(s) most commonly understood by mothers and staff and written using simple words.	

Note : If the response to S1_1 is "No" or "Don't know", indicators S1_2, S1_3, S1_4 and S1_5 are not asked.

Step 2 (Neonatal expansion)

Educate and train all staff in the specific knowledge and skills necessary to implement this policy.

Original BFHI Step 2: Train all health care staff in skills necessary to implement this policy.

<table>
<tr><th></th><th>Indicators</th><th colspan="5">Options</th></tr>
<tr><td>S2_1</td><td>S2_1 The clinical staff who have any contact with mothers and their babies cared for in the neonatal ward, receive orientation to the breastfeeding/infant feeding policy when they begin working in your neonatal ward.</td><td>None</td><td>Not many</td><td>About half</td><td>Many</td><td>All</td></tr>
<tr><td>S2_2</td><td>S2_2 The clinical staff in your neonatal ward know the importance of breastfeeding/breast milk feeding for preterm/ill infants, including benefits to the mothers.</td><td>None</td><td>Not many</td><td>About half</td><td>Many</td><td>All</td></tr>
<tr><td rowspan="3">S2_3
(Mean of 2 responses)</td><td>S2_3 The clinical staff members working in your neonatal ward for 6 months or more have received a sufficient number of hours of training to adequately support breastfeeding and lactation. (Note: The number of hours of training required may vary according to the type of clinical work).</td><td colspan="5"></td></tr>
<tr><td>S2_3a Nursing staff.</td><td rowspan="2">None</td><td rowspan="2">Not many</td><td rowspan="2">About half</td><td rowspan="2">Many</td><td rowspan="2">All</td></tr>
<tr><td>S2_3b Medical staff.</td></tr>
<tr><td>S2_4</td><td>S2_4 Part of this training includes sufficient number of hours of clinical supervision in the neonatal ward. (Note: The number of hours of supervision required may vary according to the type of clinical work).</td><td>No</td><td>Yes</td><td></td><td></td><td></td></tr>
<tr><td rowspan="5">S2_5
(Mean of 4 responses)</td><td>S2_5 The training covers:</td><td colspan="5"></td></tr>
<tr><td>S2_5a The Three Guiding Principles as stated previously.</td><td rowspan="4">No</td><td rowspan="4">Yes</td><td rowspan="4">Don't know</td><td rowspan="4"></td><td rowspan="4"></td></tr>
<tr><td>S2_5b The Ten Steps as they apply to neonatal care.</td></tr>
<tr><td>S2_5c The International Code of Marketing of Breast milk Substitutes as it applies to neonatal wards.</td></tr>
<tr><td>S2_5d How to provide support for non-breastfeeding mothers.</td></tr>
</table>

Note : If the response to S1_1 is "No" or "Don't know", indicator S2_1 is not asked.

Step 3 (Neonatal expansion)

Inform hospitalized pregnant women at risk for preterm delivery or birth of a sick infant about the benefits of breastfeeding and the management of lactation and breastfeeding.

Original BFHI Step 3: Inform all pregnant women about the benefits and management of breastfeeding.

	Indicators	Options				
S3_1	**S3_1** Your hospital has hospitalized pregnant women who are at risk of having infants admitted to your neonatal ward.	No	Yes			
S3_2	**S3_2** Hospitalized pregnant women who are at risk of having infants admitted to your neonatal ward are visited by clinical staff from your ward to offer them information about breastfeeding and lactation specific to their situation.	Never	Rarely	Half the time	Frequently	Always
S3_3	**S3_3** Your neonatal ward has a guideline describing the information about breastfeeding and lactation that the staff should provide to these women.	No	Yes			

Note : If the response to S3_1 is "No", indicators S3_2 and S3_3 are not asked and the indicators are not applicable (NA).

Step 4 (Neonatal expansion)

Encourage early, continuous and prolonged mother-infant skin-to-skin contact (Kangaroo Mother Care) without unjustified restrictions.

Original BFHI Step 4: Help mothers initiate breastfeeding within a half-hour of birth. This step was interpreted in the 2009 revision of the BFHI as:
Place babies in skin-to-skin contact with their mothers immediately following birth for at least an hour. Encourage mothers to recognize when their babies are ready to breastfeed and offer help if needed.

	Indicators	Options
S4_1	S4_1 Infants are placed in skin-to-skin contact/kangaroo position with their mothers or fathers in your neonatal ward.	No Yes
S4_2	S4_2 Your hospital has a delivery ward/room.	No Yes
S4_3 (Mean of 2 responses)	S4_3 The next statements address stable preterm infants, that is, born at 28 weeks or more of gestation without severe physiological instability associated with routine care and handling.	
	S4_3a Stable preterm infants born vaginally at 28 to 33 weeks of gestation most often initiate skin-to-skin contact with their mothers (fathers as a substitute) for the first time in your hospital/neonatal ward:<br>1. Immediately or within 5 minutes after birth.<br>2. During the first hour after birth (after the first 5 minutes but during the first hour).<br>3. During the 2nd to 24th hour of life (later than 1 hour after the birth, but during the first day of life).<br>4. After the first day.<br>5. Infants born at 28 to 33 weeks gestation are never cared for in your ward on the first days of life.	Check answer that applies
	S4_3b Stable preterm infants born vaginally at 34 to less than 37 weeks of gestation most often initiate skin-to-skin contact with their mothers (fathers as a substitute) for the first time in your hospital/neonatal ward:<br>1. Immediately or within 5 minutes after birth.<br>2. During the first hour after birth (after the first 5 minutes but during the first hour).<br>3. During the 2nd to 24th hour of life (later than 1 hour after the birth, but during the first day of life).<br>4. After the first day.<br>5. Infants born at 34 to less than 37 weeks gestation are never cared for in your ward on the first days of life.	
S4_4	S4_4 Infants are born by caesarean section performed without general anesthesia (i.e., using spinal or epidural anesthesia) in your hospital.	
S4_5	S4_5 Stable preterm infants born by cesarean section without general anesthesia at 34 to less than 37 weeks of gestation initiate skin-to-skin contact with their mothers (fathers as a substitute) **for the first time** in your hospital/neonatal ward:<br>1. Immediately or within 5 minutes after birth.<br>2. During the first hour after birth (after the first 5 minutes but during the first hour).<br>3. During the 2nd to 24th hour of life (later than 1 hour after the birth, but during the first day of life).<br>4. After the first day.<br>5. Infants born at 34 to less than 37 weeks gestation are never cared for in your ward on the first days of life.	Check answer that applies

	Indicators (continued)	Algorithm				
S4_6 (Mean of 3 responses)	S4_6 An initially unstable preterm infant is placed in skin-to-skin contact/kangaroo position in your neonatal ward as soon as the infant tolerates transfer back and forth from the mother.					
	S4_6a Infants born at less than 28 weeks gestation.	Never	Rarely	Half the time	Frequently	Always
	S4_6b Infants born at 28 to 33 weeks gestation.					
	S4_6c Infants born at 34 to less than 37 weeks gestation.					
S4_7	S4_7 Stable infants **are allowed to remain** in skin-to-skin contact/kangaroo position in your neonatal ward continuously, or for as long and as often every day as the parents are able and willing to.	No	Yes			
S4_8	S4_8 In general, how many hours per day are stable preterm infants in skin-to-skin contact/kangaroo position with their parents in your ward (please estimate the total amount of hours by adding the duration of all episodes that a stable preterm infant typically spends in skin-to-skin contact/kangaroo position on a given day)?<br>1. Less than 1 hour per day<br>2. From 1 hour to less than 2 hours per day<br>3. From 2 hours to less than 4 hours per day<br>4. From 4 hours to less than 6 hours per day<br>5. From 6 hours to less than 8 hours per day<br>6. From 8 hours to less than 12 hours per day<br>7. From 12 hours to less than 20 hours per day<br>8. 20 hours and more per day	Check answer that apply				
S4_9	S4_9 Mothers are encouraged to continue providing skin-to-skin contact/KMC for stable infants throughout their hospital stay.	Never	Rarely	Half the time	Frequently	Always

Note 1: These indicators apply to all infants admitted to your neonatal ward, whether they are breastfed or not.

Note 2: These indicators refer to all types of skin-to-skin contact (intermittent and continuous) between the mother and the preterm/low birthweight/ill infant requiring neonatal care, including skin-to-skin contact as one of the components of Kangaroo Mother Care (KMC). Skin-to-skin contact means that the infant is placed chest to chest with the parent. The infant is naked, except for a diaper (and hat and socks) to allow face, chest, abdomen, arms and legs to remain in skin-to-skin contact with the parent's chest and abdomen. The father or significant others can provide skin-to-skin/KMC as a substitute for the mother.

Note 3: If the response to S4_1 is "No", indicators S4_3, S4_5, S4_6, S4_7, S4_8 and S4_9 are not asked.

Note 4: If the response to S4_2 or S4_4 is "No", indicators S4_3 and S4_5, respectively, are not asked and the indicators are not applicable (NA).

Note 5: For indicator S4_6, if the ward does not care for unstable infants born at the gestational ages in "a", "b" or "c", they should answer "No infants" and the indicators are not applicable (NA).

Note 6: The benefits of skin-to-skin lasting at least one hour are well document. Although the optimal duration of skin-to-skin contact is not known, evidence suggest that more hours of contact is better. For the purpose of this survey, points for indicator S4_8 increase according to the number of hours of contact.

Step 5 (Neonatal expansion)

Show mothers how to initiate and maintain lactation, and establish early breastfeeding with infant stability as the only criterion.

Original BFHI Step 5: Show mother how to breastfeed and how to maintain lactation, even if they should be separated from their infants.

	Indicators	Options				
S5_1	S5_1 Breastfeeding mothers have access to breastfeeding support in your neonatal ward whenever needed.	Never	Rarely	Half the time	Frequently	Always
S5_2	S5_2 The clinical staff in your neonatal ward teach mothers how to position and attach their babies for breastfeeding.	Never	Rarely	Half the time	Frequently	Always
S5_3	S5_3 The clinical staff in your neonatal ward makes sure that infants who are able to feed at the breast, are not prevented from doing so, even during the night or during medical rounds/change of staff shifts.	Never	Rarely	Half the time	Frequently	Always
S5_4	S5_4 Mothers who plan to breastfeed/breast milk feed are offered information, support and practical help with initiation of milk production within 6 hours of their infants' birth.	None	Not many	About half	Many	All
S5_5	S5_5 Mothers who need to initiate lactation by expression are informed that the optimal strategy is to express their milk at least 7 times every 24 hours.	None	Not many	About half	Many	All
S5_6	S5_6 Mothers who are breastfeeding/breastmilk feeding or intending to do so are shown how to express their milk by hand or given printed/digital information on hand expression.	None	Not many	About half	Many	All
S5_7	S5_7 Mothers who are not exclusively breastfeeding and want to breastfeed/breastmilk feed are shown or given printed/digital information on how to use a breast pump.	Never	Rarely	Half the time	Frequently	Always
S5_8	S5_8 Infant stability is the only criterion for early initiation of breastfeeding (i.e., sucking at the breast). (A stable infant is one who responds to routine care and handling without experiencing severe apnoea, desaturations or bradycardia).	No	Yes			
S5_9	S5_9 Mothers of late preterm infants (34 to less than 37 weeks gestation) are offered the same support in the establishment of lactation and breastfeeding as those with infants born before 34 weeks.	Never	Rarely	Half the time	Frequently	Always
S5_10	S5_10 Mothers who have decided not to breastfeed are shown individually (one-on-one) how to prepare and give their babies infant formula.	Never	Rarely	Half the time	Frequently	Always

Note 1: If the hospital does not have one or more breast pumps available for mothers to use, indicator S5_7 is not applicable (NA).

Note 2: If there are no infants born at 34 to less than 37 weeks gestation that may be admitted to the ward, indicator S5_9 is not applicable (NA).

Step 6 (Neonatal expansion)

Give newborn infants no food or drink other than breast milk unless medically indicated.

Original BFHI Step 6: Same wording for the original BFHI and the Neo-BFHI.

	Indicators	Options				
S6_1	**S6_1** Infants in your neonatal ward are fed only breast milk (at the breast, expressed or donor), unless there are acceptable medical reasons to use breast-milk substitutes.	None	Not many	About half	Many	All
S6_2	**S6_2** When feasible and considering the infant's feeding tolerance, milk intake is increased before the introduction of fortifiers.	Never	Rarely	Half the time	Frequently	Always

Note 1: To be considered "only breast milk fed", infants may also receive oral rehydration solutions, drops, syrups (vitamins, minerals, medicines), or intravenous fluids. For the purpose of this survey, fortifiers are considered a medicine.

Note 2: The acceptable medical reasons to use breast-milk substitutes can be found in the following site: http://apps.who.int/iris/bitstream/10665/69938/1/WHO_FCH_CAH_09.01_eng.pdf?ua=1)

Note 3: If the neonatal ward does not use fortifiers to increase infants' intake of protein and other nutrients, indicator S6_2 is not applicable (NA).

Step 7 (Neonatal expansion)

Practice rooming-in – allow mothers and infants to remain together – 24 hours a day.

Original BFHI Step 7: Enable mothers and infants to remain together 24 hours a day.

	Indicators	Options				
S7_1	**S7_1** The mothers' presence beside their infants is unrestricted, even during emergency situations and medical rounds.	No	Yes			
S7_2	**S7_2** Mothers are allowed to be present in the same room as their infants admitted to your neonatal ward.	Never	Rarely	Half the time	Frequently	Always
	The next statements refer to mothers' possibility of sleeping close to their infants admitted to your neonatal ward.					
	**S7_3** Mothers have the possibility of sleeping in a bed in the same room as the infant during:					
	**S7_3a** Infant's whole hospital stay.					
	**S7_3b** At least 50% of the infant's hospital stay.					
	**S7_3c** At least 1 night just before the infant's discharge home.					
	**S7_4** Mothers have the possibility of sleeping in a bed in another room in your neonatal ward during:					
S7_3, S7_4 and S7_5 (Best of 9 responses)	**S7_4a** Infant's whole hospital stay.					
	**S7_4b** At least 50% of the infant's hospital stay.	None	Not many	About half	Many	All
	**S7_4c** At least 1 night just before the infant's discharge home.					
	**S7_5** Mothers have the possibility of sleeping in a bed in another area of the hospital or close to the hospital (10 minutes walking distance from infant or less) during:					
	**S7_5a** Infant's whole hospital stay.					
	**S7_5b** At least 50% of the infant's hospital stay.					
	**S7_5c** At least 1 night just before the infant's discharge home.					

Note 1: These indicators apply to all infants admitted to your neonatal ward, whether they are breastfed or not.

Note 2: For indicators S7_3, S7_4 or S7_5 if "a" is All, the rest of the indicators of Step 7 are not applicable (NA).

Note 3: For indicator S7_3, if the response to "b" is All, then "c" is not applicable (NA) and indicator S7_4 needs to be answered.

Note 4: For indicator S7_4, if the response to "b" is All, then "c" is not applicable (NA) and indicator S7_5 needs to be answered.

Note 5: For indicator S7_5, if the response to "b" is All, then "c" is not applicable (NA).

Step 8 (Neonatal expansion)

Encourage demand breastfeeding or, when needed, semi-demand feeding as a transitional strategy for preterm and sick infants.

Original BFHI Step 8: Encourage breastfeeding on demand.

	Indicators	Options				
S8_1	**S8_1** The individual infant's ability and stability – not a certain gestational/postnatal/postmenstrual age or weight – indicates when it is possible to discontinue scheduled feedings and tube feedings.	Never	Rarely	Half the time	Frequently	Always
S8_2	**S8_2** Routine supplementation with fixed volumes of milk at scheduled times (for infants who can't feed at the breast) is discontinued when the infant is able to obtain milk at the breast and replaced by a feeding plan based on the individual infant's breastfeeding behavior.	Never	Rarely	Half the time	Frequently	Always
S8_3	**S8_3** Mothers receive guidance from staff on how to recognize their infants' feeding cues and behavioral changes in order to help determine when it is appropriate to breastfeed.	Never	Rarely	Half the time	Frequently	Always
S8_4	**S8_4** Medications are administered and procedures (such as blood tests, eye exams, medical imaging) are scheduled so as to cause the least possible disturbance to breastfeeding.	Never	Rarely	Half the time	Frequently	Always

Step 9 (Neonatal expansion)

Use alternatives to bottle feeding at least until breastfeeding is well established, and use pacifiers and nipple shields only for justifiable reasons.

Original BFHI Step 9: Give no artificial teats or pacifiers (also called dummies or soothers) to breastfeeding infants.

	Indicators	Options				
S9_1	**S9_1** Mothers who are breastfeeding, or intending to do so, feed their infants in your neonatal ward without using bottles.	Never	Rarely	Half the time	Frequently	Always
S9_2	**S9_2** The clinical staff avoid introducing bottles to infants if the mother's goal is to exclusively breastfeed.	Never	Rarely	Half the time	Frequently	Always
S9_3	**S9_3** Pacifiers are only used in your neonatal ward for justifiable reasons (pain relief or when infant cannot be comforted at breast, skin-to-skin or with other methods).	Never	Rarely	Half the time	Frequently	Always
S9_4	**S9_4** Breastfeeding mothers are informed about the above justifiable reasons for use of pacifiers in your neonatal ward.	Never	Rarely	Half the time	Frequently	Always
S9_5	**S9_5** Nipple shields are only recommended when breastfeeding problems persist after having received skilled breastfeeding support to resolve the underlying breastfeeding problems.	Never	Rarely	Half the time	Frequently	Always

Note 1: If infants in the neonatal ward do not use pacifiers, indicators S9_3 and S9_4 are not applicable (NA).
Note 2: If the neonatal ward does not have nipples shields for mother's use, indicator S9_5 is not applicable (NA).

Step 10 (Neonatal expansion)

Prepare parents for continued breastfeeding and ensure access to support services/groups after hospital discharge.

Original BFHI Step 10: Foster the establishment of breastfeeding support groups and refer mother to them on discharge from the hos

	Indicators	Options				
S10_1	**S10_1** Your neonatal ward can discharge infants directly to their home.	No	Yes			
S10_2	**S10_2** Mothers are informed of the resources available in the hospital or in the community in case they have questions about infant feeding after their return home.	Never	Rarely	Half the time	Frequently	Always
S10_3	**S10_3** Your neonatal ward collaborates with mother support groups that provide breastfeeding/infant feeding support.	No	Yes			
S10_4	**S10_4** The hospital discharge for infants who have been cared for in your neonatal ward is planned in collaboration with the family and community health services.	Never	Rarely	Half the time	Frequently	Always
S10_5	**S10_5** Mothers are encouraged to have their babies seen soon after discharge, either at the hospital or in the community, by skilled breastfeeding support persons who can assess feeding and give any support needed.	Never	Rarely	Half the time	Frequently	Always

Note : If the response to S10_1 is "Never", indicators S10_2 to S10_5 are not asked and are not applicable (NA).

International Code
of Marketing of Breast-milk Substitutes and relevant World Health Assembly resolutions.

	Indicators	Options				
C_1	Code_1 Your hospital/healthcare facility refuses free or low-cost supplies of breast-milk substitutes.	No	Yes	Don't know		
C_2	Code_2 Your neonatal ward refrains from promoting breast-milk substitutes, bottles, teats, or pacifiers in your neonatal ward.	No	Yes			
C_3	Code_3 Your neonatal ward refrains from giving pregnant women, mothers and their families any marketing materials, samples or gift packs that include breast-milk substitutes, bottles/teats, pacifiers or other equipment or coupons.	No	Yes			
C_4	Code_4 Employees of manufacturers or distributors of breast-milk substitutes, bottles, teats, or pacifiers are prohibited from any contact with pregnant women or mothers in your neonatal ward.	No	Yes	Don't know		
C_5	Code_5 Your neonatal ward refuses free gifts, non-scientific literature, materials or equipment, money or support for in-service education or events from manufacturers or distributors of breast-milk substitutes, bottles, teats, or pacifiers.	No	Yes			
C_6	Code_6 Your neonatal ward keeps infant formula cans and pre-prepared bottles of formula out of view unless in use.	No	Yes			
C_7	Code_7 The clinical staff working in your neonatal ward understand why it is important not to give any free samples or promotional materials from formula companies to mothers.	None	Not many	About half	Many	All

Note: The indicators refer to the Code as it applies to the neonatal ward.

Additional information for all levels of care

Written protocols and guidelines

Questionnaire completed on:

	Question	Options
P_1	P_1 In the previous statements we asked you about current practices in your neonatal ward. In this section, we would like to know which of the following points are included/addressed in written protocols and guidelines at your hospital or neonatal ward.	
	1. Parents should be integrated as essential partners in the care of their infants.	Check all answers that apply
	2. The neonatal ward is open to parents 24 hours a day, 7 days a week.	
	3. Skin-to-skin/KMC is promoted for all preterm and ill infants whether they are breastfed or not.	
	4. Infant stability is the only criterion for initiation of breastfeeding (sucking at the breast).	
	5. Newborns are given no food or drink other than breast milk (at breast, expressed or donor) unless there are acceptable medical reasons.	
	6. Infants who are able to obtain some milk at the breast are breastfed on demand or with a semi-demand strategy (depending on the infants' ability).	
	7. Appropriate and safe use of alternative methods to bottle feeding.	
	8. Justifiable reasons for use of pacifiers.	
	9. Other breastfeeding related points (please describe).	
	10. None of these points are addressed in written protocols, guidelines or standards.	
	11. We have no written protocols, guidelines or standards related to lactation, breastfeeding or infant feeding.	

Additional information for all levels of care

Description of the neonatal ward

	Characteristic of the neonatal ward	Options
N_2	N_2 Questionnaire answered by:	
	Head Nurse<br>Breastfeeding Staff<br>Medical doctor<br>Other	Check answer that applies
N_3	N_3 Chose the highest level of neonatal care in your ward:	
	Level of care 1<br>Level of care 2<br>Level of care 3a<br>Level of care 3b<br>Level of care 3c	Check answer that applies
N_4	N_4 Which does best describe your neonatal ward/unit:	
	Exclusive neonatal<br>Mixed neonatal-maternity/postpartum/nursery.<br>Mixed neonatal-pediatric<br>Other	Check answer that applies
N_7	N_7a Do you have an early discharge program for preterm infants with nasogastric tube in order to establish breastfeeding at home?	No Yes
	N_7b Do you have a Kangaroo Mother Care program for preterm infants with early discharge and follow-up?	No Yes
N_8	N_8 Does your unit have access to banked or donor human milk?	No Yes
N_9	N_9 Infants admitted to your neonatal ward are transferred from other hospitals.	
	None or Not Many<br>About half<br>Many or All	Check answer that applies
N_10	N_10 Please indicate the type of staff that have direct responsibility for assisting mothers with infants admitted to your neonatal ward with lactation, breastfeeding and infant feeding.	
	Nurses, nursing assistants and midwifes working primarily in your neonatal ward/units.<br>Lactation consultants.<br>Physicians (neonatologists, paediatricians, obstetricians or other).<br>Dieticians/Nutritionists.<br>Occupational therapists/Speech therapists.<br>Lay support persons/peer counsellors.<br>Other.<br>No staff responsible.	Check all answer that apply
N_12	N_12 In your hospital, are there any committees for breastfeeding/infant feeding, BFHI, Neo-BFHI, Kangaroo Mother Care, or HIV infant feeding?	No Yes

	Characteristic of the neonatal ward (continued)	Options
N_5	N_5 Authorized/Designated capacity of infants in the ward (number).	Write number
N_6	N_6 Number of infants in your neonatal ward yesterday.	Write number
N_11	N_11 Please describe the approximate number of:	
	N_11a Nurses, nursing assistants and midwifes working primarily in your neonatal ward/units.	Write number
	N_11b Lactation consultants.	Write number
	N_11c Physicians (neonatologists, paediatricians, obstetricians or other).	Write number

Additional information for all levels of care

Information about Baby-friendly designation and comments about the survey

	Additional information about the BFHI	Options
A_1	A_1 Has your hospital been fully accredited "Baby-friendly", that is has completed an external evaluation process and has been accredited Baby-friendly by the national authority responsible for the BFHI?	No Yes
	A_1a Indicate the year of the 1st time it was fully accredited (received the award).	Write year
	A_1b Indicate the year of the last re-accreditation (if ever).	Write year
A_2	A_2 Has your neonatal ward been fully accredited "Baby-friendly", that is, has completed an external evaluation process SEPARATE FROM THE ONE FOR MATERNITY WARDS and accredited Baby-friendly by the national authority responsible for the BFHI?	No Yes
	A_2a Indicate the year of the 1st time it was fully accredited (received the award).	Write year
	A_2b Indicate the year of the last re-accreditation (if ever).	Write year
A_3	A_3 Which statement best describes the situation of your neonatal ward? Assume that it is possible to obtain a Baby-friendly designation for neonatal wards. Your answer does not constitute any commitment from you or your organization:	
	We don't have enough information about the Baby-friendly initiative for neonatal wards to decide if we intend to seek accreditation.	Check answer that apply
	Even if we received the information, we do not intend to register to start the process to obtain Baby-friendly accreditation for neonatal wards, or not before 2020.	
	We would like to register to start the process to obtain Baby-friendly accreditation for neonatal wards by 2017…	
	…by 2018	
	…by 2019	
	We have already registered to start the process to obtain Baby-friendly accreditation for neonatal wards.	
	Our neonatal ward is already fully accredited "Baby-friendly" and we want to maintain the accreditation.	
	Our neonatal ward is already fully accredited "Baby-friendly" and we do not want to maintain the accreditation.	
	Don't know	
A_4	A_4 If you have comments, check "Yes" and there will be a space to write any comments you may want to make about your answers to the survey, or the expansion of the Baby-friendly Hospital Initiative to neonatal wards. Otherwise, check "No".	No Yes
	If yes to A_4 **Comments given by your unit (text limited to 500 characters):**	
