# Supplementary figures and images for: Compliance with the “Baby‐friendly Hospital Initiative for Neonatal Wards” in 36 countries
Source: Matern Child Nutr. 2018 Oct 12;15(2):e12690. doi: 10.1111/mcn.12690 (PMC6586157; doi:10.1111/mcn.12690)

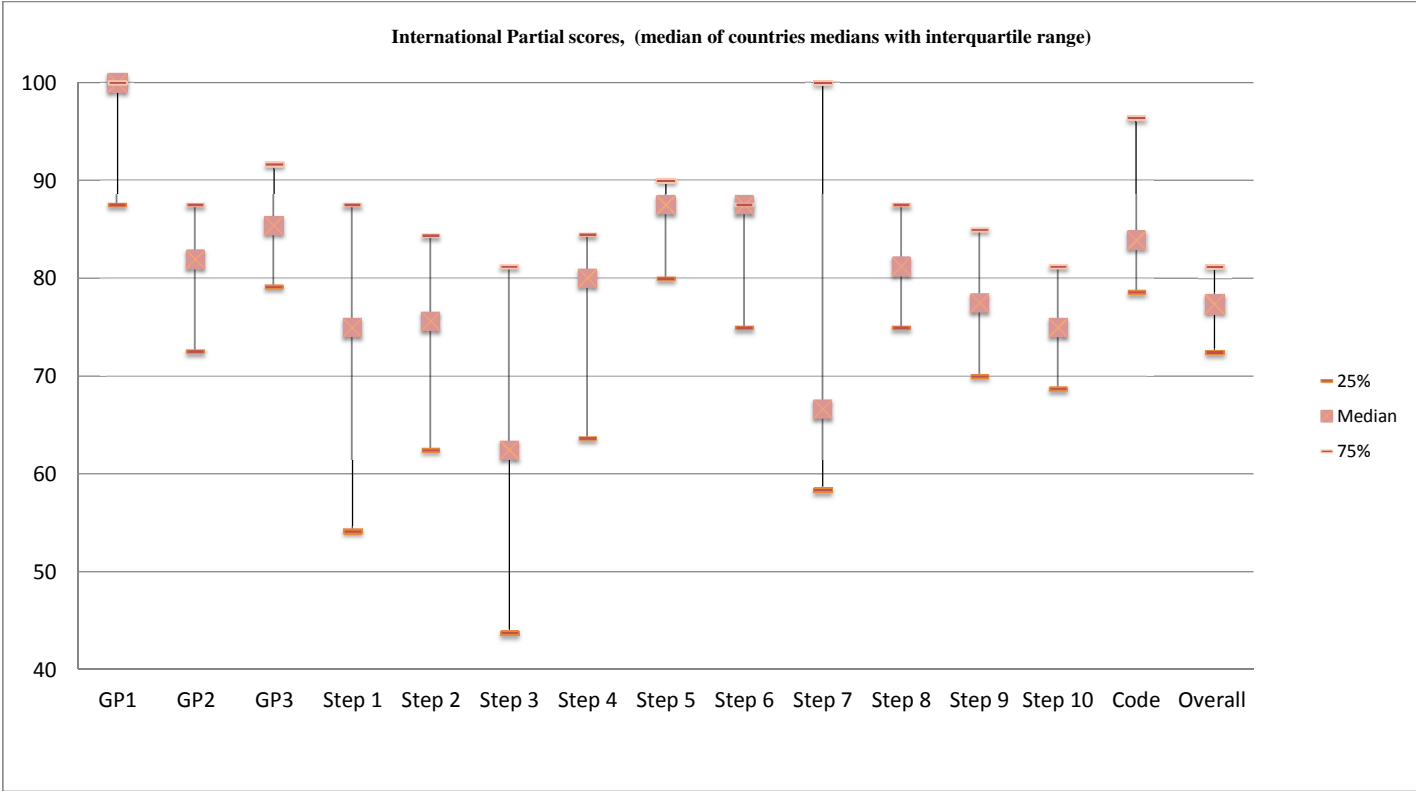

Supplement: Supplementary file 2 — Data S2 International Partial Scores. Medians with interquartile range [file MCN-15-e12690-s002.pdf]
